# CD24 Expression May Play a Role as a Predictive Indicator and a Modulator of Cisplatin Treatment Response in Head and Neck Squamous Cellular Carcinoma

**DOI:** 10.1371/journal.pone.0156651

**Published:** 2016-06-08

**Authors:** Vishnu Modur, Pooja Joshi, Daotai Nie, K. Thomas Robbins, Aziz U. Khan, Krishna Rao

**Affiliations:** 1 Department of Medical Microbiology, Immunology and Cell Biology, SIU, Springfield, Illinois, United States of America; 2 Division of Hematology and Oncology, Department of Internal Medicine, SIU, Springfield, Illinois, United States of America; 3 Department of ENT – Otolaryngology, SIU, Springfield, Illinois, United States of America; 4 Southern Illinois University School of Medicine, Springfield, Illinois, United States of America; 5 Simmons Cancer Institute at SIU, Post Office Box 19678, Springfield, Illinois 62794–9678, United States of America; Ludwig-Maximilians University, GERMANY

## Abstract

Platinum-based therapy is most often used to treat advanced cases of head and neck cancers, but only a small fraction of the patient population responds to cisplatin, with a median survival time of less than a year. Although gene signatures and molecular etiology of head and neck cancers have been previously described, none of them are predictive indicators of cisplatin treatment response in particular. Therefore, currently, there is a lack of clinically employable predictive indicators of the disease beyond HPV status to specifically predict patients' response to platinum-based therapy. It beckons a substantial effort to look for predictive indicators of cisplatin treatment response. In this regard, CD24 expression level appears to be a significant molecular phenotype of cisplatin-resistant residual cells in laryngeal carcinoma lines. CD24 expression level directly affects cisplatin sensitivity and affects the expression of critical apoptotic, stem and drug resistance genes. A relatively small retrospective patient tumor analysis suggests that CD24 high tumors go on to show an unfavorable response to cisplatin treatment. Overall, based on the strength of further analysis, CD24 presents a strong rationale to be utilized as a predictive indicator to stratify head and neck cancer patients for platinum-based therapy. It also provides a rationale for using CD24 as a therapeutic adjuvant target along with standard cisplatin therapy.

## Introduction

Head and neck squamous cell carcinoma (HNSCC) is a cluster of biologically similar cancers that originate from the mucosal squamous epithelial lining of the upper aerodigestive tract. HNSCC is the sixth most frequently occurring cancer worldwide. Although early-stage HNSCC have high cure rates, up to 50% of patients present with advanced disease [1]. Patients presenting with advanced disease of HNSCC are associated with a high mortality rate. Despite advances in therapy, close to 50% of advanced HNSCC tumors relapse within the first 24 months of treatment [2] [3]. Currently, cisplatin-based chemotherapy is the most commonly used treatment for advanced cases, but only about 10–35% responds to cisplatin, with a median survival time of 6–12 months [4]. Presently, there is a lack of clinically employable predictive indicators of the disease beyond HPV status to specifically predict patients' response to platinum-based therapy [5]. Hence, to look for potential predictors of cisplatin treatment response to classify patients who may or may not benefit from platinum-based therapy is paramount.

Prognostic indicators such as histologic appearance, lymph node involvement, and presence of distant metastasis have limited value in predicting response to a particular treatment. Breast cancer expression of HER2, for example, can predict the effectiveness of Herceptin therapy. Similarly, in HNSCC, HPV+ status portends a favorable response to chemoradiation. However, the lack of any other suitable predictive indicator to platinum-based treatment response in HNSCC poses a clinical hurdle. Residual cells by definition are more resistant to cisplatin and can be surveyed as a therapy-induced enrichment of molecular markers that earmark an already existing resistant population in a tumor. Therefore, the residual cell population can be an invaluable resource to look for pre-existing predictive indicators of cisplatin treatment response [6]. The CSC hypothesis asserts that these residual cells are functionally, TPCs (tumor propagating cells), which are a naturally chemo-resistant, self-renewing fraction of the tumor [4] [7]. It is a well-established fact that CD44 and CD24 are often co-utilized along with other tertiary markers to isolate TPCs in various cancers. CD24 is a small heavily glycosylated glycosylphosphatidylinositol-linked cell surface protein, a ligand for P-selectin, broadly expressed on B-cells and neuroblasts. It is expressed in hematological malignancies as well as in a wide array of solid tumors. In recent years, CD24 gene has raised considerable interest in tumor biology and poor treatment outcome. CD24 expression causes the acquisition of multiple cellular properties associated with tumor growth and metastasis [8]. Recent studies have identified that the positive selection of CD24 selects for cancer stem cells in several cancers, including pancreatic cancer [9] colorectal cancer [10], liver [11], and ovarian cancer [12]. On the other hand, the negative selection of CD24 also has resulted in the selection of cancer stem cells in some other cancers such as breast and prostate [13] [14]. These findings indicate that while CD44 may broadly mark for TPCs in various cancers, the co-expression of CD24 or the lack of it distinctively marks for TPCs in a tissue-specific manner. It is an especially noteworthy feature of CD24 that it marks for an unfavorable outcome in cancers from various tissues, like larynx [15], lung [16], Ovary [17], breast [13] [18] and prostate [14]. Ergo, CD24 not only is a tissue type specific TIC/CSC marker but a valuable indicator for poor treatment outcome in multiple cancer types. Therefore, under the purview of TPCs/CSC cells, CD24 is an attractive marker to explore the possibility of it predicting cisplatin treatment response in HNSCC. In this study, we chose three laryngeal carcinoma cell lines, namely, UM-SCC-10B, UM-SCC-15s, and UM-SCC-74B. UM-SCC-15s is a resistant line derived from UM-SCC-10B on repeated exposure to cisplatin in culture. UM-SCC-74B is a laryngeal carcinoma line obtained from a second surgery post chemoradiation treatment. We initially aimed to assess the levels of CD24 expression in the three chosen laryngeal carcinoma lines to see if there is any correlation between surface level CD24 expression and the corresponding cisplatin sensitivity of the lines. We then probed whether modulation of the levels of CD24 affected cisplatin resistance in the three laryngeal carcinoma lines and the expression of various pro-survival genes involved in cisplatin resistance. Ultimately, this study addresses the expression of CD24 in human xenografts in nude mice and in human tumor samples to observe any correlation between CD24 levels and the eventual outcome of cisplatin treatment response.

## Materials and Methods

The IRB committee approved the use of 25 human head and neck tumor samples for our study of CD24 in cisplatin treatment response. Name of IRB: Springfield Committee for Research Involving Human Subjects (SIU School of Medicine) Name of the tissue bank: Simmons Cancer Institute at Southern Illinois University School of Medicine. Tissue Bank Protocol (08-112/ 12–177). All specimens were collected after obtaining written informed consent from the patient or their legally authorized representative. SIU School of Medicine, Laboratory Animal Care and Use Committee (LACUC) approved the animal protocol involving athymic nude mice. The protocol included xenograft assays and drug treatment. Mice were monitored every 3 days. Humane endpoints were used for mice displaying moribund signs such as extreme weight loss, loss of appetite, ulceration of the tumor. Animals were euthanized by CO2 inhalation followed by cervical dislocation.

### Cell Culture and Cisplatin preparation

The human squamous cellular carcinoma lines UM-SCC-10B and its cisplatin resistant version UM-SCC-15s were provided to us by Dr. Howell from Departments of Medicine, University of California, San Diego, La Jolla, USA. The human squamous cellular carcinoma line, UM-SCC-74B, was provided to us by Dr.Thomas E. Carey from University of Michigan. UM-SCC-10B and UM-SCC-15s were grown in complete RPMI (Roswell Park Memorial Institute) medium; UM-SCC-74B was grown in complete Dulbecco’s Modified Eagle’s Medium (DMEM). Both cell culture media contained 2 mM L-glutamine, 1% nonessential amino acids, 1% Penicillin-Streptomycin (Lonza, Walkersville, MD) and 10% FBS (fetal bovine serum), in a humidified chamber of 5% CO2 at 37°C. All cell lines were tested for mycoplasma, using the Mycoplasma Detection Kit (Southern Biotech, Birmingham, AL).

### Cisplatin and IC-50 determination

Cisplatin (*cis*-Diammineplatinum(II) dichloride) (ACROS Organics, NJ, USA) was prepared as a stock of 3.33mM in 1X PBS. The IC-50 value of cisplatin in the head and neck cell lines was determined by seeding 5000 cells/well in a 96 well plate for 24 hours followed by the addition of cisplatin in increasing doses from 0–400mM for 48 hours of exposure. The percent cell viability was calculated by the addition of a cell viability detection reagent–PrestoBlue (Invitrogen, NY, USA). After 2 hours of incubation the change in the color of the substrate was detected by Fluoroskan Ascent (Thermo Scientific).

### Cell growth assay

The seven head and neck cell lines were counted and seeded at the concentration of 5000 cells/well in different 96 well plates. Percent viability of cells from each line was calculated by the addition of a cell viability detection reagent–PrestoBlue (Invitrogen, NY, USA). After 2 hours of incubation the change in the color of the substrate was detected by Fluoroskan Ascent (Thermo Scientific). This process was carried out each day up to day 6. A graph was plotted using the increase in number of viable cells each day as measured in each line.

### Radiation resistance assay

The seven head and neck cell lines were counted and seeded at the concentration of 5000 cells/well in different 96 well plates. After 24 hours of incubation a plate of each line was exposed to radiation from 0–3 Gy. The plates exposed to 0 rads were used as controls. After exposure to radiation the cells were incubated for 48 hours. Then percent viability of cells from each line was calculated by the addition of a cell viability detection reagent–PrestoBlue (Invitrogen, NY, USA). After 2 hours of incubation the change in the color of the substrate was detected by Fluoroskan Ascent (Thermo Scientific).

### Preparation of RNA, cDNA and RT-PCR

RNA was isolated from cells using Qiagen kit. The cDNA was prepared by reverse transcription using the RevertAid First Strand cDNA synthesis kit (Thermo Scientific), and used as a template for RT-PCR (GoTaq qPCR Promega kit). RT-PCR reaction was run on an ABI cycler using primer sequences listed below. Threshold cycles were normalized relative to B-Actin expression. Error bars represent the standard deviation of the mean. The primers used are given in Table 1 below.

**Table 1:** Real time PCR primers to probe gene expression in lines with modified CD24 expression levels.

Gene	Primer
**B-ACTIN Forward**	**GATCATTGCTCCTCCTGAGC**
**B-ACTIN Reverse**	**GGCAAGGGACTTCCTGTAAC**
**CD24 Forward**	**CCCACGCAGATTTATTCCAG**
**CD24 Reverse**	**GACTTCCAGACGCCATTTG**
**CCND1 Forward**	**CACGGACTACAGGGGAGTTT**
**CCND1 Reverse**	**TCTGTTCCTCGCAGACCTCC**
**BCLx1 Forward**	**CCTGCCTGCCTTTGCCTAA**
**BCLX1 Reverse**	**CACAAAAGTATCCCAGCCGC**
**MDR3 Forward**	**AGAGGGGCTGAAGCCTGATA**
**MDR3 Reverse**	**ACCATCGAGAAGCACTGTCC**
**MDR1 Forward**	**GAGCCTACTTGGTGGCACAT**
**MDR1 Reverse**	**TCCTTCCAATGTGTTCGGCA**
**RAD50 Forward**	**CGAAGTACCTATCGTGGACAAG**
**RAD50 Reverse**	**GATCGTCCTCGCATATCCAAG**
**NBS1 Forward**	**AGACCAACTCCATCAGAAACTAC**
**NBS1 Reverse**	**AATGAGGGTGTAGCAGGTTG**
**L1CAM Forward**	**TGCTCATCCTCTGCTTCATC**
**L1CAM Reverse**	**TCCTCGTTGTCACTCTCCA**

### Western Blotting (WB)

This was performed as described previously [19]. Antibodies used included rabbit monoclonal antibody against β-actin (Cell Signaling Technology, MA, USA) and mouse monoclonal against CD24 –Clone SN3 (Thermo Scientific, MA, USA).

### CD24 siRNA transfection

CD24 siRNA and control duplex, ‘5-GATTTATTCCAGTGAAACA-3’, and ‘5-GGTCTCACTCTCTCTTCTGCATCTCTACT-3’, were purchased from Applied Biological Materials (Richmond, Canada). CD24 and control siRNAs were transfected into a growing culture of 293T cells with the aid of packing vectors and Lipofectamine transfection reagent (Invitrogen, NY, USA). The resulting viral supernatant after 48 hours was collected and introduced to a growing culture of UM-SCC-10B and UM-SCC-15s with the aid of polybrene reagent (Millipore, MA, USA). Stable UM-SCC-10B CD24-KD and UM-SCC-15s CD24-KD (knockdown) clones were obtained by Puromycin selection (3μg/μl) in vitro.

### CD24 Overexpression

CD24 mammalian expression vector (EX-G0006-Lv105-10) and a control vector (EX-NEG-Lv105) were purchased from Genecopoeia (Rockville, MD, USA). CD24 mammalian expression vector and control vectors were transfected into a growing culture of 293T cells with the aid of packing vectors and Lipofectamine transfection reagent (Invitrogen, NY, USA). The resulting viral supernatant after 48 hours was collected and introduced to a growing culture of UM-SCC-74B with the aid of polybrene reagent (Millipore, MA, USA). Stable UM-SCC-74B CD24-UP (upregulation) clones was obtained by Puromycin selection (3μg/μl) in vitro.

### Anti-CD24 therapy with cisplatin

CD24 antihuman antibody (Thermo Scientific, MA, USA) was treated against CD24-high head and neck cancer cell lines at 1:500 dilutions in 1X PBS for 48 hours prior to further treatment with cisplatin.

### Immunohistochemistry

The mouse anti-human CD44 (mouse IgG2b, BD Pharmingen, CA, USA) and mouse anti-human CD24 (Thermo Scientific, MA, USA) primary antibodies were used. Immunostaining was performed using the diaminobenzidine (DAB) substrate method (Cell Signaling Technology, MA, USA) according to the manufacturer’s protocol. Before specific staining, unspecific antigenic sites were blocked with normal calf serum. Sections were then incubated with the respective primary antibody for 1 hour at room temperature (RT) followed by incubation with horse radish peroxidase single stain detection reagent (10 minutes at RT). Later, sections were washed with water for 5 minutes. Specific peroxidase activity was visualized with DAB working solution (contains DAB chromogen concentrate and DAB diluent). Counterstaining was performed with Mayers hematoxylin and eosin. A scoring scale of relative intensity of positive staining was used. A weak staining was given a score of 1, moderate staining was scored 2 and strong staining was given a score of 3. Assessment of the intensity of positive staining was performed across the entire sample in a blinded fashion upon light microscopy by two investigators and is given as the mean of both scores assessed.

### Flow cytometry

Head and neck squamous cell carcinoma cells UM-SCC-10B, UM-SCC-15s and UM-SCC-74B were stained with mouse anti-human CD44 (APC conjugated, BD Pharmingen, CA, USA) or mouse anti-human CD24 (PE-Cy-7 conjugated, BD Pharmingen, CA, USA). Isotype controls APC mouse IgG2b k and PE-Cy-7 mouse IgG2a k were used to set the gates. Live cells were gated using PI staining (BD Pharmingen, CA, USA). The antibody treated cells were washed in PBS before the flow cytometry analysis using BD FACS Aria^™^ II (Becton Dickinson, CA, USA).

### Orosphere Generation

Culture cells were harvested in trypsin-EDTA and carefully resuspended in DMEM/F-12 medium (Lonza, Walkersville, MD) supplemented with 1% N2 supplement, 2% B27 supplement, 20ng/ml b-FGF-2 and 20ng/ml EGF (GIBCO Life Technologies, NY, USA). Around 5000 cells were counted and plated in each well of a six-well ultra-low-attachment plate (Corning, Lowell, MA). Orospheres were assayed 7 to 14 days after cell plating. Orospheres were counted manually under a light microscope, and experiments were done in triplicates.

### Tumor Samples

Head and neck primary tumor specimens from 25 patients who received cisplatin treatment were obtained after the necessary IRB approval. Tissue procurement from the tissue bank and screening process was approved by Simmons Cancer Institute at SIU. They also verified all the frozen tumor tissue samples and de-identified them before the process began.

### CD24 knockdown xenograft assay and cisplatin treatment

To initiate the experiment on tumor growth and cisplatin resistance, 5 × 10^6^ tumor cells (UM-SCC-10B and UM-SCC-10B CD24 KD; UM-SCC-15s and UM-SCC-15s CD24 KD) were injected subcutaneously on the ventral sides of 4–5-week-old athymic mice. Xenograft size was measured every 3 days and tumor volume was determined as (length × width^2^)/2. After 7 days, 16 mice each harboring tumors on both ventral sides cells were randomly separated into two groups of 8: vehicle (PBS) and cisplatin (5 mg/kg/week). Both vehicle and cisplatin was administrated by intraperitoneal injection weekly. The size of the tumor was followed for 21 days. The end point of the study is the end of 3 rounds of cisplatin/vehicle treatment. The significant difference in p-value for each group at the end of 21 days after the initiation of treatment was calculated by Student’s t-test and defined by as P<0.05.

### Statistical Analysis

Statistical analysis was performed using GraphPad Prism 6.0 version. The fishers T-test was used to compare the correlation between CD24 expression, CD44 expression and poor outcome to cisplatin therapy. Student’s T-test was used to calculate the significance in quantitative PCR experiments. Statistical analyses were performed in cell growth rate assay, tumor volume measurements and radiation sensitivity assay. Overall, all data presented as standard error or mean. In all tests, p values of ≤0.05 were considered significant and p values >0.05 and <0.1 considered to represent a trend.

## Results

### Stem-like cells positively marked by CD44 are not necessarily resistant to cisplatin

**Fig 1:**
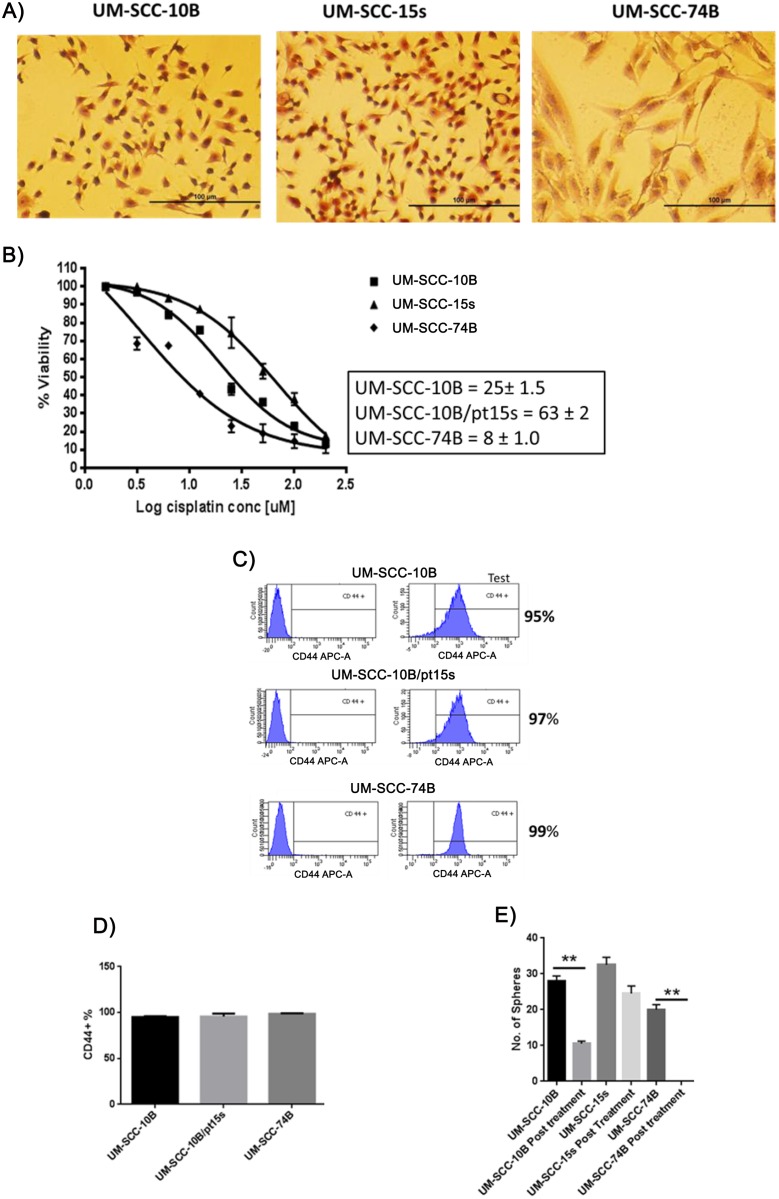
No Correlation between CD44% and cisplatin sensitivity. A) Hematoxylin and Eosin staining of three laryngeal carcinoma cell lines. B) Cisplatin IC-50 calculated for the three laryngeal carcinoma lines. C) Flow cytometry analysis of membrane bound CD44 on the three laryngeal carcinoma lines and D) graphical representation of CD44% with standard error of mean (t-test, n = 3). E) Graph of the number of orospheres formed with standard error of mean (t-test, n = 3).

To investigate whether CD44 percentage correlates with cisplatin resistance in laryngeal carcinoma; we chose three laryngeal carcinoma cell lines namely, UM-SCC-10B, UM-SCC-15s (the cisplatin resistant pair of UM-SCC-10B) and UM-SCC-74B (Fig 1A). These three lines show varying degrees of cisplatin resistance (UM-SCC-10B–25μM, UM-SCC-15s–63μM and UM-SCC-74B–8μM) (Fig 1B). Once the cisplatin IC-50 values of the three HNSCC lines were determined, analysis of the three cell lines with CD44 monoclonal antibody in a flow cytometer was performed. It demonstrated that CD44 percentage is overwhelmingly high (~97%) in all these three HNSCC lines irrespective of their varying cisplatin sensitivities (Fig 1C and 1D). Orosphere formation is a test that quantifies the self-renewing capacity of the cells and by doing so, it provides evidence of the ‘stem’ property of these cancer cells. The three HNSCC lines were capable of forming orospheres when they were untreated with cisplatin but upon cisplatin treatment at IC-50 dose for each of these three lines, only UM-SCC-10B and UM-SCC-15s were capable of forming orospheres and not UM-SCC-74B (Fig 1E and 1F). These results show that not all CD44+ cells are stem-like and resistant to chemotherapy. Although the three lines are overwhelmingly CD44+, the distinct behavior of UM-SCC-74B in its inability to form orospheres after an IC-50 dose of cisplatin is evidence enough that CD44 alone is insufficient in marking chemoresistant cells.

### CD24 positivity shows linear relationship with cisplatin resistance

**Fig 2:**
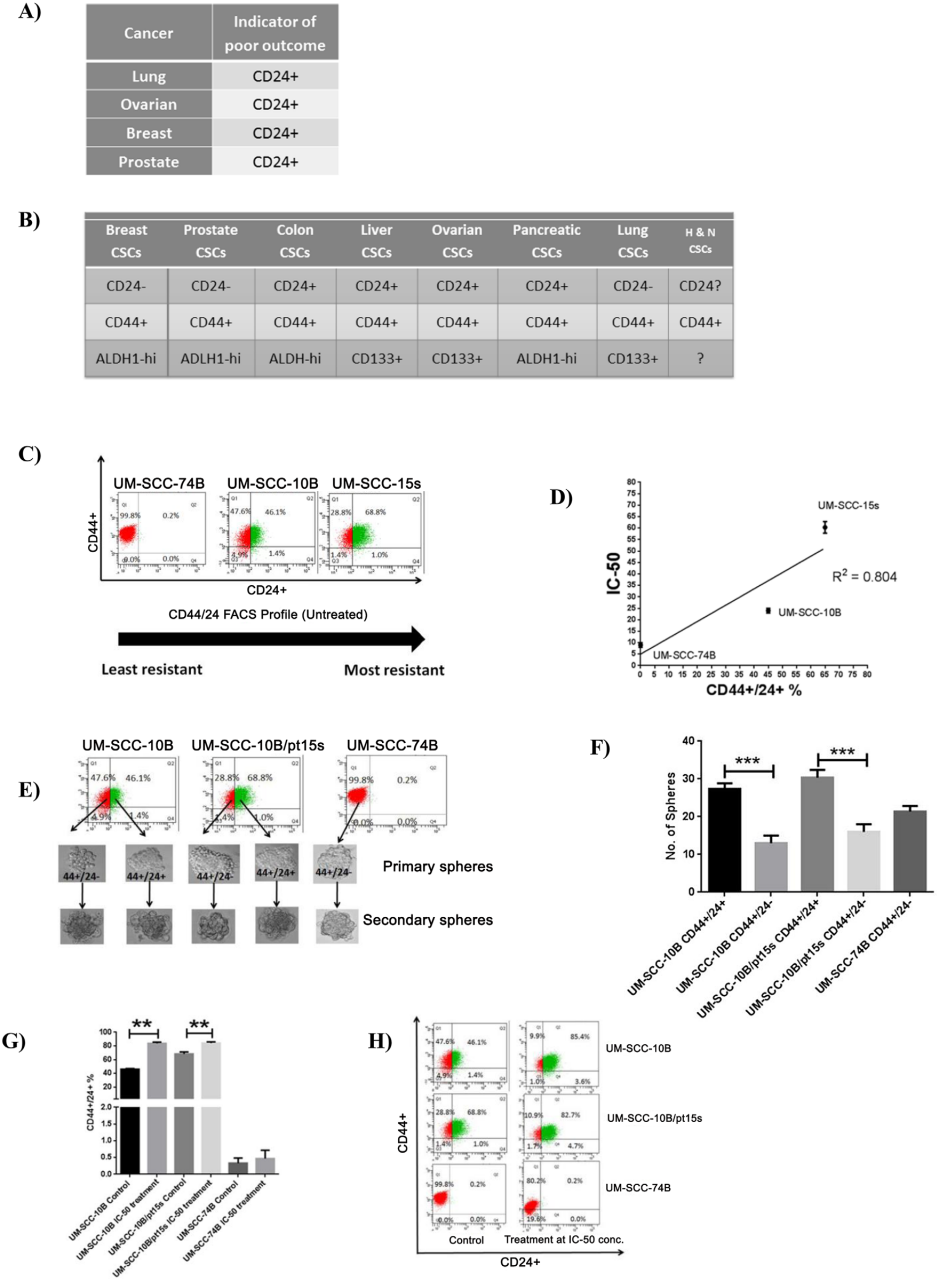
Correlation between CD24% and cisplatin sensitivity. A) Tabulation showing that CD24+ is an indicator of poor outcome in multiple cancers. B) Tabulation showing that the positive or negative expression of CD24 is the discerning factor in isolating cancer stem cells/ tumor propagating cells in various cancers. C) Flow cytometry analysis of membrane bound CD24 and CD44 of the three laryngeal carcinoma lines show an increasing fraction of CD24% and D) Graphical representation of CD24% with R^2^ = 0.804 showing a positive linear correlation with increasing cisplatin resistance in the three laryngeal carcinoma lines. E) Primary and secondary orospheres formed by the CD24+ (green) and CD24– (red) fractions. F) Graph of the number of orospheres formed as shown in E) with standard error of mean (t-test, n = 3). G) CD24+ residual fraction increase in proportion as it remains viable even after an IC-50 dose of cisplatin treatment. H) Graph with standard error of mean (t-test, n = 3) of the percent increase in proportion of the residual viable CD24+ fraction after an IC-50 dose of cisplatin treatment.

Now that the previous experiment clearly demonstrated the inability of CD44 alone in marking a cisplatin resistant stem population and its lack of correlation with cisplatin IC-50 values, another important stem related marker was to be looked at. CD24 is often used along with CD44 in demarcating a CSC population in multiple cancers. Therefore, CD24 formed the focus of further experimentation to look for its ability to mark a cisplatin resistant stem population and the correlation of its expression with the cisplatin IC-50 values in these HNSCC lines. CD24 is an indicator of poor outcome in various cancers (Fig 2A) [20]. It is also a stem marker in various cancers (Fig 2B) [21]. Its role in head and neck cancer stem cells is still quite unclear. To investigate whether CD24 percentage correlates with cisplatin resistance in these three laryngeal carcinoma lines, we performed a flow cytometry analysis (Fig 2C). The percentage of CD24+ cells shows a linear relationship with the IC-50 values on the three laryngeal carcinoma lines (Fig 2D). Also, both, CD24+, and CD24- fractions were capable of forming primary and secondary generation orospheres (Fig 2E). However, the CD24+ cells in UM-SCC-10B and UM-SCC-15s could form orospheres in significantly greater numbers as compared to CD24- cells (Fig 2F). The CD24+ cells in these two lines were enriched as a residual resistant fraction of cells on treatment with an IC-50 dose of cisplatin (Fig 2G and 2H). When 50% of UM-SCC-10B and UM-SCC-15s cells were killed by cisplatin treatment, a highly self-renewing, resistant CD24+ cells remained viable. This provides considerable evidence that CD24 marks for a cisplatin resistant population in these HNSCC lines.

### Modulation of CD24 expression affects cisplatin sensitivity in laryngeal carcinoma lines

**Fig 3:**
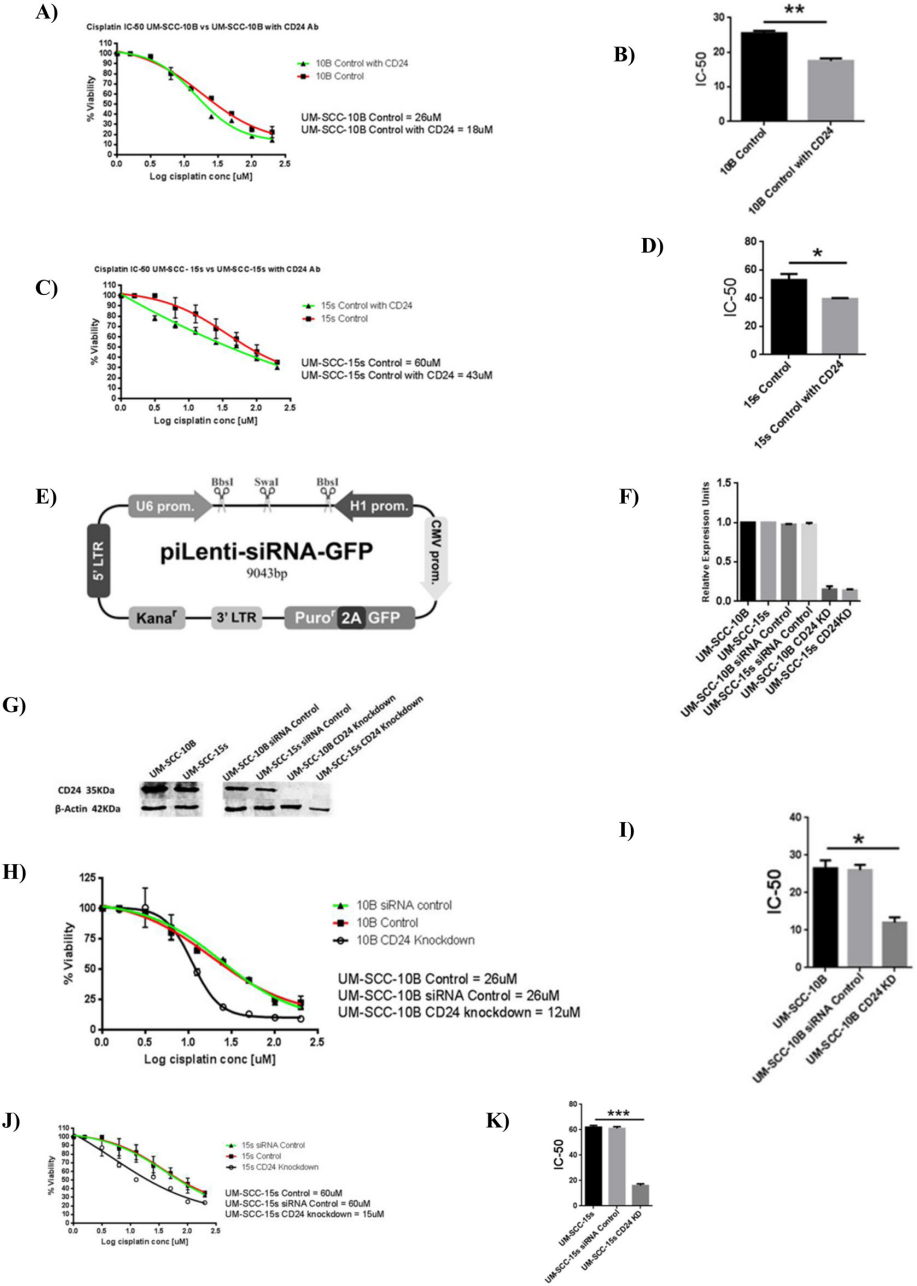
CD24 plays a functional role in determining cisplatin sensitivity of UM-SCC-10B and UM-SCC-15s. A) Cisplatin IC-50 values of CD24 antibody treated UM-SCC-10B and UM-SCC-10B without antibody treatment B) Cisplatin IC-50 value of UM-SCC-10B drops significantly on CD24 antibody treatment, standard error of mean (t-test, n = 3). C) Cisplatin IC-50 values of CD24 antibody treated UM-SCC-15s and UM-SCC-15s without antibody treatment. D) Cisplatin IC-50 value of UM-SCC-15s drops significantly on CD24 antibody treatment, standard error of mean (t-test, n = 3). E) siRNA Lentivector map purchased from Applied biological materials (Canada). F) RNA level confirmation of CD24 knockdown by targeted siRNA treatment. G) Protein level confirmation of CD24 knockdown by targeted siRNA treatment. H) Cisplatin IC-50 values of CD24 knocked down UM-SCC-10B and UM-SCC-10B control. I) Cisplatin IC-50 value of CD24 knocked down UM-SCC-10B drops significantly as compared to the parental UM-SCC-10B, standard error of mean (t-test, n = 3). J) Cisplatin IC-50 values of CD24 knocked down UM-SCC-15s and UM-SCC-15s control. K) Cisplatin IC-50 value of CD24 knocked down UM-SCC-15s drops significantly as compared to the parental UM-SCC-15s, standard error of mean (t-test, n = 3).

**Fig 4:**
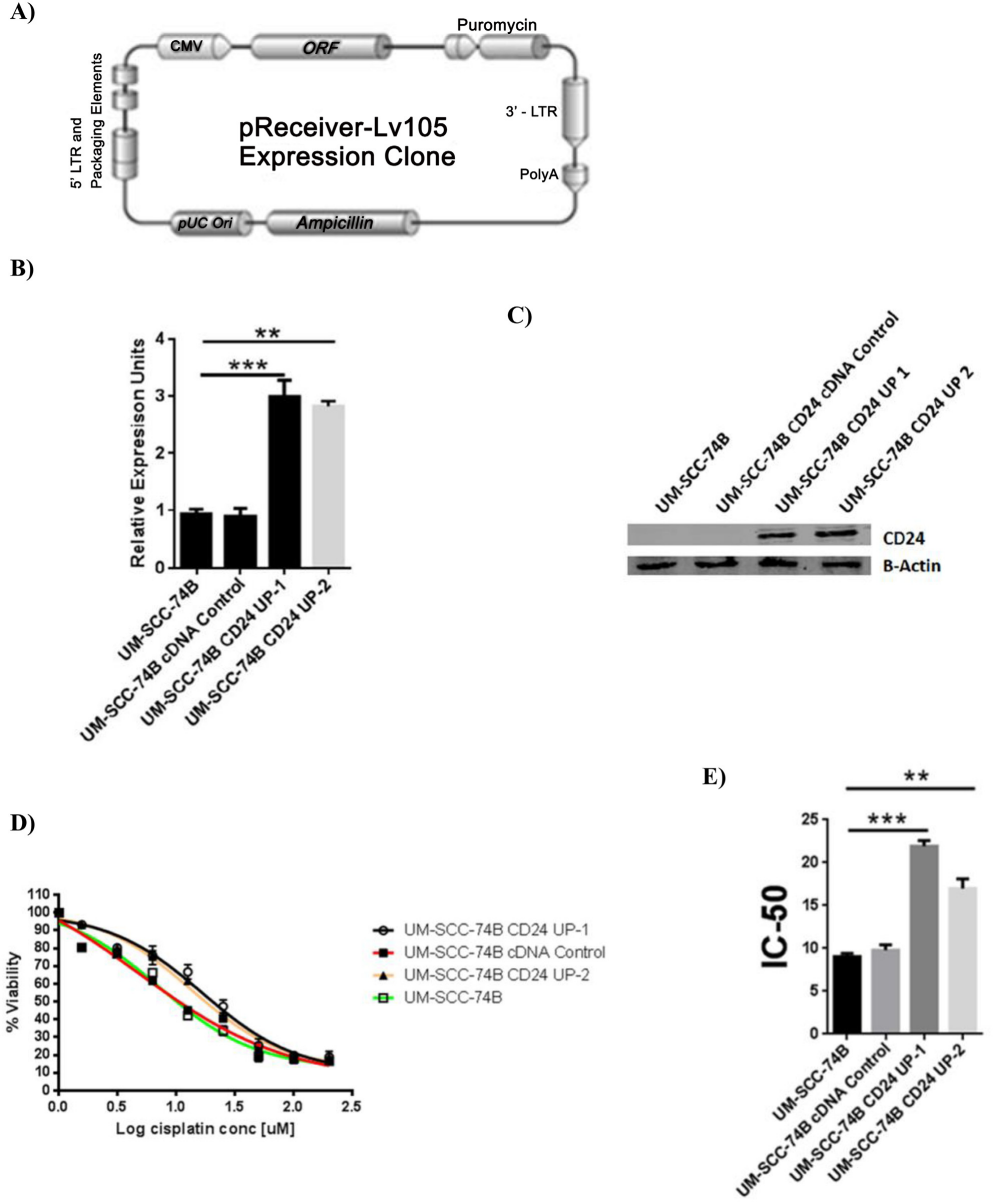
CD24 plays a functional role in determining cisplatin sensitivity of UM-SCC-74B. A) CD24 expression Lentivector map purchased from GeneCopoeia (US). B) RNA level confirmation of CD24 up-regulation in UM-SCC-74B, standard error of mean (t-test, n = 3). C) Protein level confirmation of CD24 up-regulation in UM-SCC-74B. D) Cisplatin IC-50 value CD24 overexpressing UM-SCC-74B rises significantly as compared to the parental UM-SCC-74B. E) Graph showing cisplatin IC-50 value of CD24 overexpressing UM-SCC-74B rises significantly as compared to the parental UM-SCC-74B, standard error of mean (t-test, n = 3).

**Fig 5:**
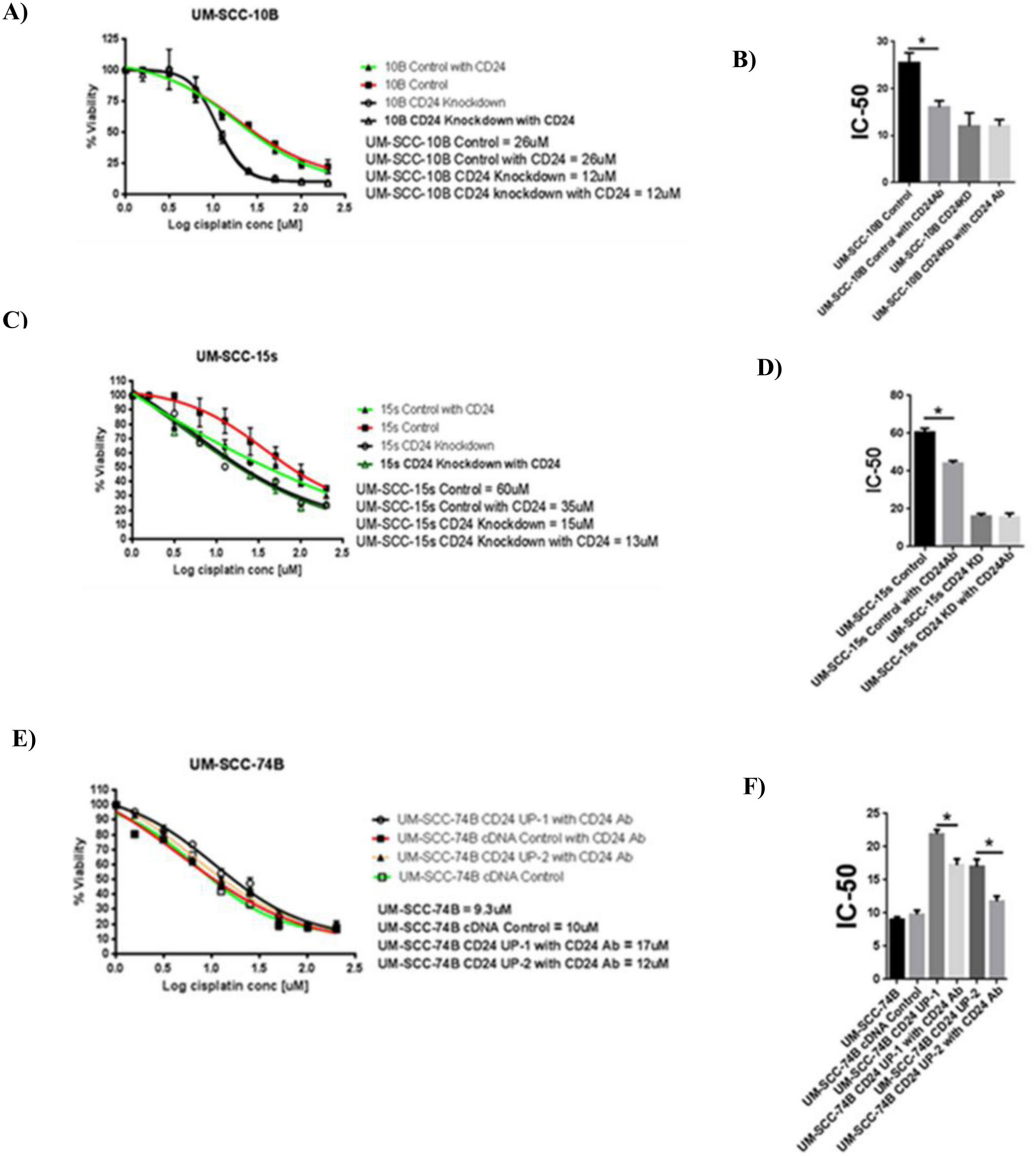
Comparison between CD24 antibody treatment and CD24 expression manipulation. A) and B) Comparison of varying cisplatin IC-50 values in UM-SCC-10B on CD24 antibody treatment, CD24 knockdown variant as compared with the control, standard error of mean (t-test, n = 3). C) and D) Comparison of varying cisplatin IC-50 values in UM-SCC-15s on CD24 antibody treatment, CD24 knockdown variant as compared with the control, standard error of mean (t-test, n = 3). E) and F) Comparison of varying cisplatin IC-50 values in UM-SCC-74B on CD24 antibody treatment, CD24 overexpression variants as compared with the control, standard error of mean (t-test, n = 3).

The flow cytometry results clearly show the composition of the residual cells to be heavily CD24+. First, anti-CD24 antibody treatment lead to significant decrease in the IC-50 values of UM-SCC-10B and UM-SCC-15s (Fig 3A–3D). This result confirmed that CD24 had a role to play in conferring cisplatin resistance. Decreased cisplatin resistance by antibody treatment against CD24 provided a strong rationale for creating stable CD24 knockdowns of UM-SCC-10B and UM-SCC-15s (Fig 3E). On confirming the knockdown of CD24 in UM-SCC-10B and UM-SCC-15s (Fig 3F and 3G), cisplatin IC-50 values were determined (Fig 3H and 3I). The CD24 knockdown versions of UM-SCC-10B and UM-SC-15s showed a significant drop in their cisplatin IC-50 values compared to their CD24 intact parental lines (Fig 3J and 3K). In the case of UM-SCC-74B –a CD24 negative cell line, CD24 was stably overexpressed (Fig 4A). On confirming the up-regulation of CD24 in UM-SCC-74B (Fig 4B and 4C), cisplatin IC-50 values were determined. Results indicate that cisplatin IC-50 values increased significantly upon up-regulation of CD24 in the two CD24 overexpressing clones of UM-SCC-74B (Fig 4D and 4E). Following on similar lines with that of parental UM-SCC-10B and UM-SCC-15s, the resulting elevated IC-50 value of the CD24 overexpressing UM-SCC-74B line was mitigated to a significant extent by treatment with an anti- CD24 monoclonal antibody (Fig 5A–5F).

### Modulating CD24 expression leads to variations in the expression of various pro-survival genes

**Fig 6:**
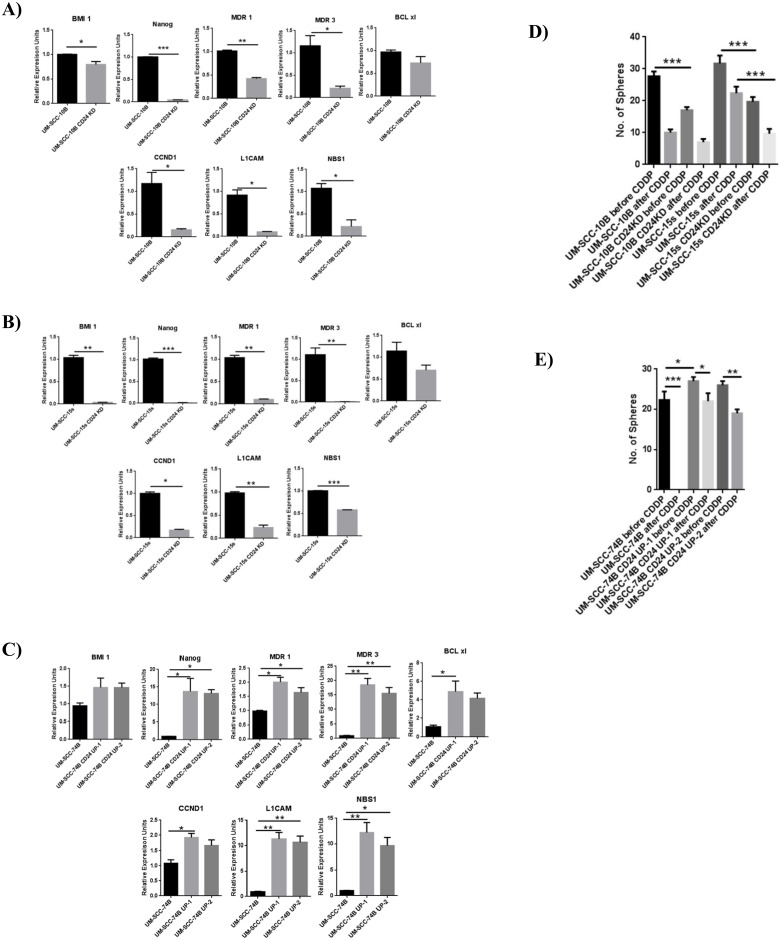
Modulation of CD24 expression directly affects the gene expression of various pro-survival genes critical in cisplatin resistance. A) and B) Down regulation of CD24 in CD24 rich UM-SCC-10B and UM-SCC-15s leads to significant down regulation of critical pro-survival genes, standard error of mean (t-test, n = 3). C) Up regulation of CD24 in CD24 deficient UM-SCC-74B leads to significant up regulation of critical pro-survival genes, standard error of mean (t-test, n = 3). D) Graph of the number of orospheres formed by the CD24 knockdown variants of UM-SCC-10B and UM-SCC-15s before and after an IC-50 dose of cisplatin treatment, standard error of mean (t-test, n = 3). E) Graph of the number of orospheres formed by the CD24 overexpressing variants of UM-SCC-74B before and after an IC-50 dose of cisplatin treatment, standard error of mean (t-test, n = 3).

Having demonstrated that CD24 expression has a strong linear correlation with cisplatin IC-50 values and a direct functional effect on cisplatin sensitivity, quantitative PCR further showed that modulating the expression level of CD24 in UM-SCC-10B, UM-SCC-15s and UM-SCC-74B lead to significant changes in expression of various genes related to cisplatin resistance (Fig 6A–6C). Stem-related genes involved in self-renewal such as BMI 1 and NANOG; multiple drug resistance genes such as MDR1 and MDR3; cell cycle progression, proliferation and double strand DNA break repair genes like CCND1, L1CAM and NBS1 are expressed at low levels in CD24 knockdown lines. On the other hand, these genes are upregulated in CD24 overexpressing versions of UM-SCC-74B. Another interesting outcome of modulating CD24 expression levels in these three laryngeal carcinoma lines is it affects the sphere forming/self-renewing ability. CD24 knockdown clones of UM-SCC-10B and UM-SCC-15s as compared to their parental lines form much smaller and fewer spheres before cisplatin treatment, but after cisplatin treatment, they lose the ability to form full-fledged spheres in high numbers (Fig 6D and 6E). However, CD24 overexpressing UM-SCC-74B clones not only form full-fledged spheres before cisplatin treatment, but they retain the ability form similar sized spheres even after cisplatin treatment unlike its parental line, confirming the crucial impact that CD24 has in maintaining viability and overall drug resistance (Fig 6F and 6G).

### Modulating CD24 expression leads to variations in growth rate but has no effect on radiation sensitivity

**Fig 7:**
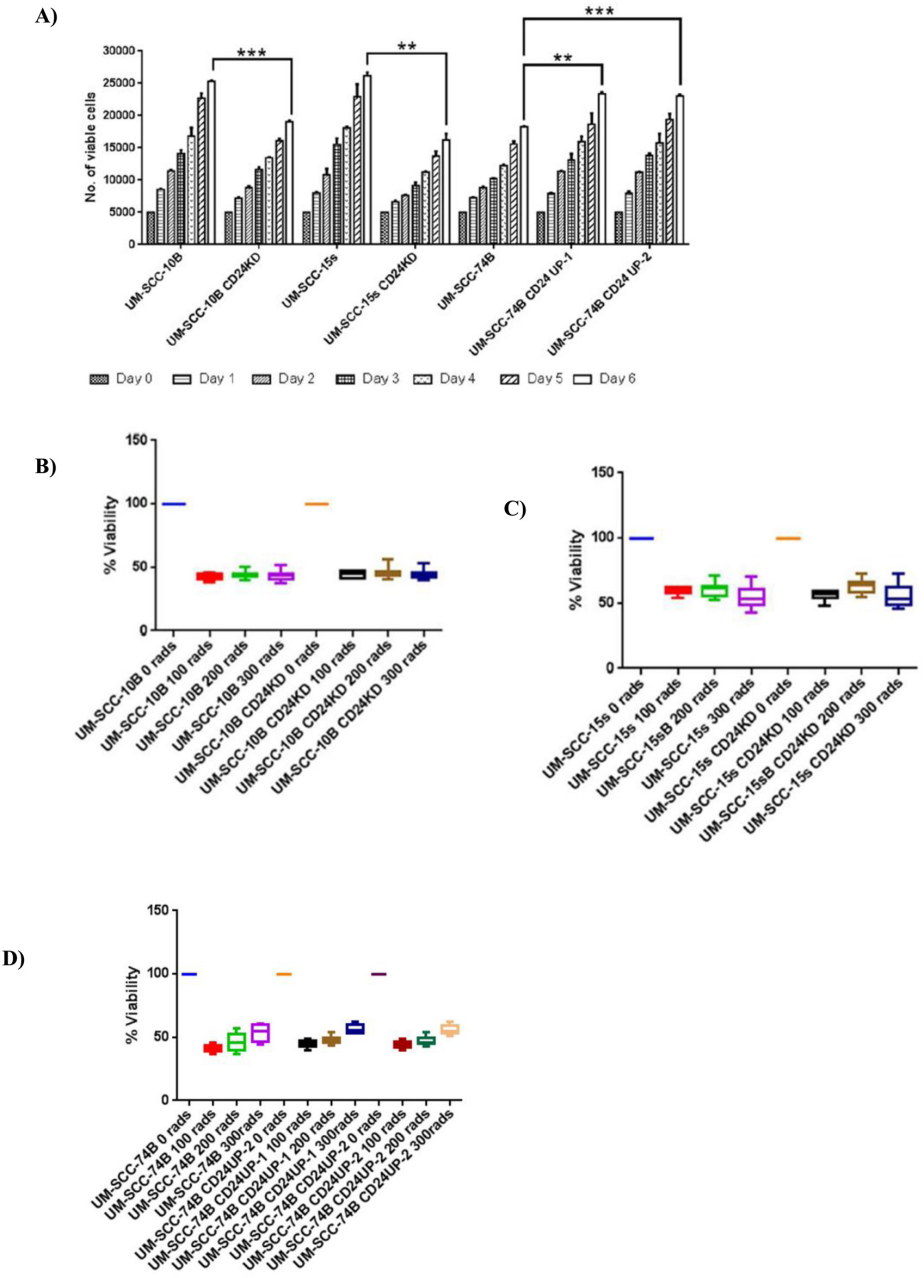
CD24 expression and its effect on growth rate and radiation sensitivity. A) Graph showing cell growth of HNSCC parental lines and their corresponding CD24 expression modulated lines for a period of six days starting at 5000 cells/well on day 0, standard error of mean (t-test, n = 3). B) Graph showing percentage of viable cells of UM-SCC-10B parental line and its corresponding CD24 expression modulated line exposed to radiation from 0 to 3 Gy. The viable cell percentage was calculated after a 48 hour recovery period, standard error of mean (t-test, n = 3). C) Graph showing percentage of viable cells of UM-SCC-15s parental line and its corresponding CD24 expression modulated line exposed to radiation from 0 to 300 rads. The viable cell percentage was calculated after a 48 hour recovery period, standard error of mean (t-test, n = 3). D) Graph showing percentage of viable cells of UM-SCC-74B parental line and its corresponding CD24 expression modulated line exposed to radiation from 0 to 300 rads. The viable cell percentage was calculated after a 48 hour recovery period, standard error of mean (t-test, n = 3).

Having established the fact that modulating CD24 expression in HNSCC lines has a direct bearing on cisplatin sensitivity, self-renewing ability and has a linear correlation with cisplatin resistance it is essential to delve into the effect of CD24 on cellular growth rate and in turn radiation resistance. Therefore, cell growth rate and radiation resistance of CD24 modulated lines, and their parental lines were assessed. Altering the expression level of CD24 in UM-SCC-10B, UM-SCC-15s, and UM-SCC-74B changes cellular growth rate significantly (Fig 7A). More precisely, knockdown of CD24 in UM-SCC-10B and UM-SCC-15s leads to significantly slower growth rates in UM-SCC-10B CD24KD and UM-SCC-15s CD24KD. Upregulation of CD24 in UM-SCC-74B lead to a significant increase in growth rates, as observed in UM-SCC-74B CD24UP1 and UM-SCC-74B CD24UP2. Nevertheless, modulation of CD24 expression in UM-SCC-10B, UM-SCC-15s and UM-SCC-74B shows no significant effect on their radiation sensitivity. Radiation sensitivities measured at 1, 2 and 3 Gy for each of pair namely, UM-SCC-10B and UM-SCC-10B CD24KD, UM-SCC-15s and UM-SCC-15s CD24KD, UM-SCC-74B and UM-SCC-74B CD24UP2 shows no statistical difference (Fig 7B–7D). However, intermediate and long-term effects of radiation on CD24 modulated lines were not determined. Putting together, these results highlight CD24 to be a fairly specific indicator of cisplatin treatment response and a valuable therapeutic target of cisplatin resistance.

### High expression of CD24 correlates with unfavorable cisplatin treatment outcome

**Fig 8:**
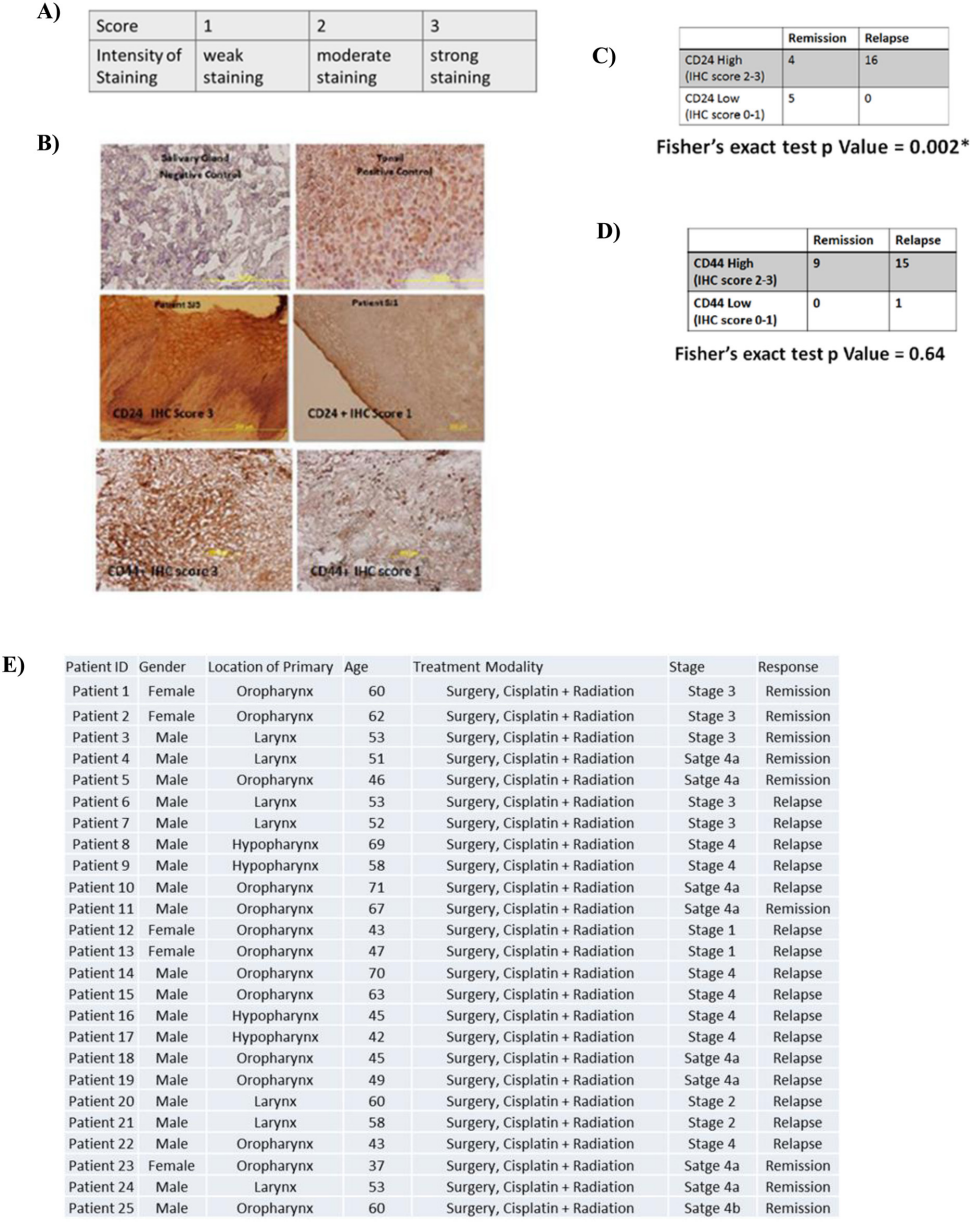
CD24 expression and its correlation with cisplatin treatment outcome. A) Scale used to score IHC sections for CD24 and CD44 expression B) Representative images of controls, CD24+ and CD44+ tissue head and neck tumor sections. C) Tabulation of the total number of patient samples (Fisher’s exact test, n = 25) scored for CD24 expression and statistically correlated with cisplatin treatment response. D) Tabulation of the total number of patient samples (Fisher’s exact test, n = 25) scored for CD44 expression and statistically correlated with cisplatin treatment response. E) Tabulation of the clinical parameters of the 25 patient samples.

Having established that CD24 specifically affects cisplatin sensitivity of these HNSCC lines and arguably has no bearing on their early radiation sensitivity profile, we intended to study the expression pattern of CD24 and compare it with the prominent CSC marker CD44 in HNSCC tumor sections. We conducted a retrospective analysis on 25 head and neck tumor samples obtained from patients who later underwent cisplatin treatment. We scored the expression of CD24 and CD44 in these samples (Fig 8A). Our results show that the expression level of CD24 alone was capable of indicating an unfavorable cisplatin treatment response (Fig 8B–8E). CD44, an established CSC marker of HNSCC proves to be incapable of predicting cisplatin treatment response. This suggests that although CD44 may mark for a stem-like population, CD44 alone marks for may not necessarily contribute towards an unfavorable cisplatin treatment response, whereas, CD24 staining intensity can discern the possibility of an unfavorable treatment response suggesting that CD24 and the population of cells it marks for is truly cisplatin resistant. Although we have used a small patient cohort in this study, we obtained a statistically significant correlation between CD24 staining intensity and unfavorable cisplatin treatment outcome. Further work in a much larger patient cohort will firmly establish the significance of CD24 as a predictive indicator for cisplatin resistance in head and neck cancers.

### CD24 Knockdown induced tumors in athymic mice respond favorably to cisplatin treatment

**Fig 9:**
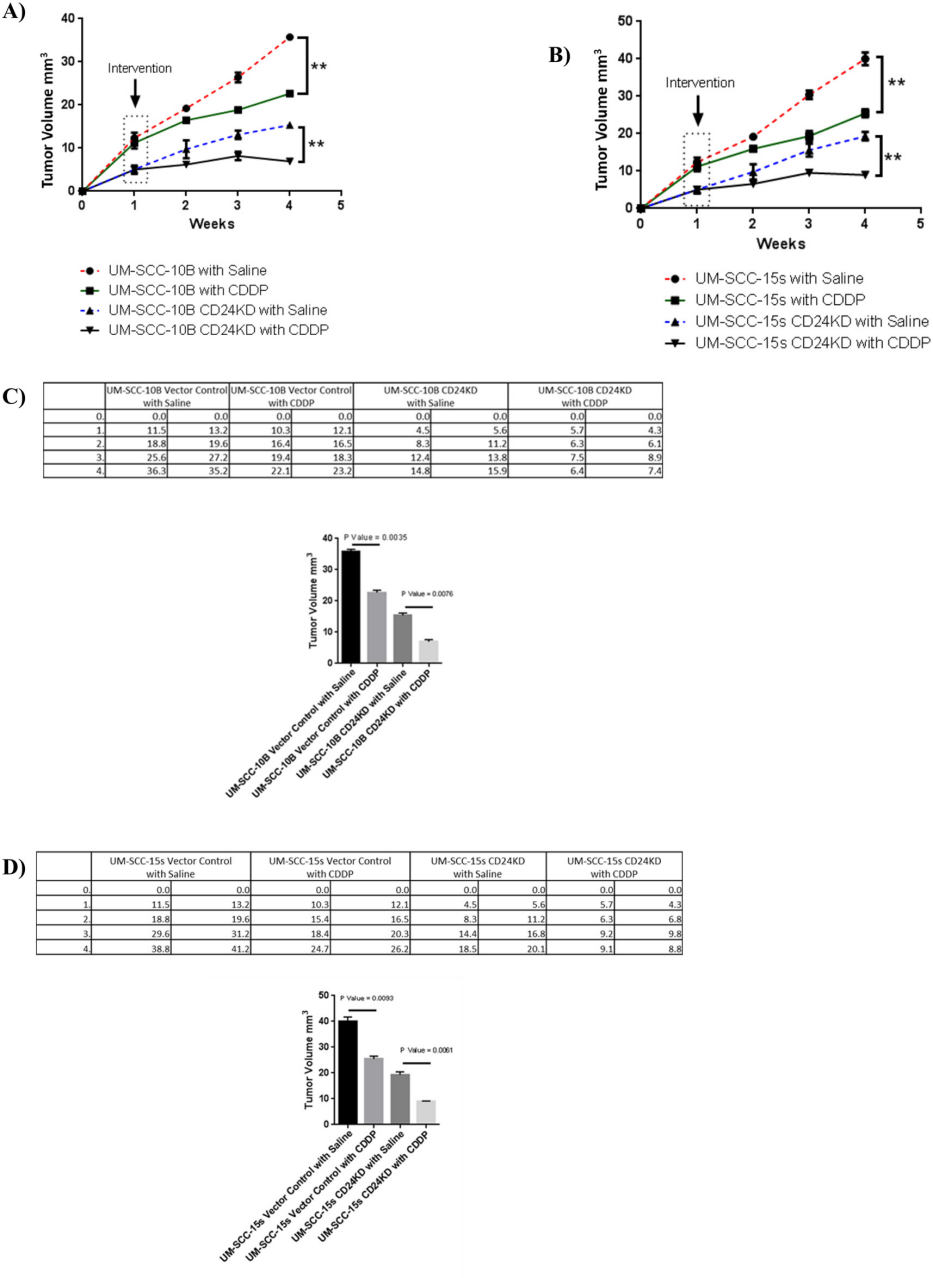
CD24 expression and its correlation with cisplatin treatment outcome. A) Tumor volumes of UM-SCC-10B vector control and its CD24 knockdown clone UM-SCC-10B CD24KD treated with Saline (Control) and Cisplatin (Test), standard error of mean (t-test, n = 8). B) Tumor volumes of UM-SCC-15s vector control and its CD24 knockdown clone UM-SCC-15s CD24KD treated with Saline (Control) and Cisplatin (Test), standard error of mean (t-test, n = 8). C) Tabulation tumor volumes measured for UM-SCC-10B and UM-SCC-10BCD 24KD; graph of the final volume of the tumors and their statistical measure (standard error of mean). D) C) Tabulation tumor volumes measured for UM-SCC-15s and UM-SCC-15s CD24KD; graph of the final volume of the tumors and their statistical measure (standard error of mean).

On underscoring the significance of utilizing CD24 as a predictive indicator of cisplatin treatment response, an in vivo experiment to test CD24 as a therapeutic target in cisplatin treatment becomes the logical step forward. The in vivo experiment will also bring the study to a full circle starting from recognizing CD24 as a marker of cisplatin resistant population in vitro to screening for CD24 in pre-cisplatin treatment patient tumor samples, to treating tumors induced with CD24 modulated cell lines with cisplatin treatment. To initiate the in vivo experiment, 5 million cells of vector control lines of UM-SCC-10B, UM-SCC-15s and their CD24 knockdown versions UM-SCC-10B CD24KD and UM-SCC-15s CD24KD were injected to induce tumors subcutaneously in athymic mice. On comparing the tumor volumes of vector control parental lines and their CD24 knockdown clones after a period of three weeks of either vehicle treatment (Saline) and cisplatin treatment, it was evident that UM-SCC-10B CD24KD and UM-SCC-15s CD24KD make much smaller tumors compared to their parental lines. The evidently smaller tumors of UM-SCC-10B CD24KD and UM-SCC-15s CD24KD (Fig 9A–9D) respond favorably to cisplatin treatment as compared to their corresponding vehicle treated controls.

## Discussion

As stated by the cancer stem cell hypothesis, a stem-like population becomes enriched upon chemotherapy due to their distinct survival advantage of enhanced self-renewing capacity. Recently, this notion has gained further traction with experimental proofs in a few cancer types [5]. The TPCs in HNSCC was first described on the basis of CD44 expression. CD44+ HNSCC cells are capable of initiating tumors that demonstrably replicate the original tumor heterogeneity in nude mice, as well as self-renewal after serial passaging in vivo [20]. However, CD44– HNSCC cells are incapable of doing the same [12]. CD44 expresses, albeit in diluted proportions, not only in malignant or benign but even in normal epithelia of the head and neck [22]. This fact argues that CD44 alone is insufficient in earmarking HNSCC TPCs [23] [21]. In fact, about 95% of the three laryngeal carcinoma lines in this study (UM-SCC-10B, UM-SCC-15s, and UM-SCC-74B) is overwhelmingly CD44+ underscoring the need for ancillary marker(s) to pinpoint residual cells/TPCs.

The CD24 gene encodes a highly glycosylated cell surface protein anchored to the membrane by phosphatidylinositol linkage [24]. CD24 is an oncogene, overexpressed in multiple human malignancies [17] [13]. Recent studies suggest that CD24 may be a negative tumor propagating marker within breast cancers [2]. Intriguingly, a large volume of evidence has shown that CD24+ tumor cells are TPCs for pancreatic cancer [9], Cholangiocarcinoma [25] and colon cancer [10]. In this study, we have shown that CD24 positivity marks for a residual cisplatin resistant population in laryngeal carcinoma lines. We have also shown that CD24+ cells maintain high self-renewing capability—a critical stem-like feature in the cisplatin resistant fraction of these cell lines. Evidently, blocking CD24 by a monoclonal antibody treatment in CD24-rich cell lines resulted in lowering cisplatin resistance. Cisplatin resistance was also significantly lowered on knockdown of CD24 expression in CD24-rich cell lines. On the contrary, CD24 overexpressing variants of a CD24-low cell line raised its cisplatin resistance. The positive correlation between the expression levels of CD24 and those of various pro-survival genes critical in mustering cisplatin resistance casts light on the vast scale of effects that CD24 expression has in maintaining the viability of laryngeal carcinoma cells. We also found a positive correlation between CD24 expression in head and neck tumor samples and unfavorable cisplatin treatment response retrospectively in a small cohort. An in vivo xenograft study in athymic mice revealed the susceptibility of tumors generated from CD24 knockdown clones of UM-SCC-10B and UM-SCC-15s towards intra-peritoneal cisplatin injection. CD24 knockdown HNSCC lines also generated much smaller tumor burden in the same time period as its vector control lines. These results suggest a crucial role of CD24 in affecting cisplatin sensitivity in HNSCC. However, the exact nature of CD24 expression and its downstream mechanism of action remain unknown and needs to be further elucidated. We hypothesize that CD24 could be a crucial controller of lipid rafts by bringing in other membrane-bound signaling molecules in proximity to initiate signaling cascade. More specifically, CD24 could act from the cell membrane through BMI1 and/or Nanog to control the expression levels of MDR genes, CCND1 and DNA repair genes like L1CAM and NBS1 to combat cisplatin-induced toxicity and damage to the cancer cells. CD24 could execute its functions by being a controller of lipid rafts, i.e., by bringing in upstream signaling complexes in proximity to initiate a signaling cascade. Hence, CD24 can act on some key signaling molecules and affect the transcription of genes involved in survival and resistance to platinum-induced toxicity. Therefore, the expression levels of CD24 can indicate the levels of sensitivity to cisplatin one can expect in a tumor. The possible reason for CD24 not contributing to radiation resistance could stem from the additional factors required by cells in displaying radiation resistance. Apart from DNA repair genes being actively transcribed, a low fidelity of DNA polymerase is crucial in contributing to radiation resistance. CD24-high cells may not have a reduced fidelity of DNA polymerase leading to segregation of chemoresistance and radioresistance we see in our experiments. Also, there could be multiple stem-like clonal subpopulations within a single tumor which have varying degrees of resistance to platinum and radiotherapy. CD24 may essentially mark for platinum-resistant sub-population which doesn't necessarily overlap a great deal with a radio-resistant subpopulation. We do have to highlight the fact that the main focus of this study has not been about radiation resistance. We have not checked intermediate and late effects of CD24 modulation on radiation resistance in these lines. Our conclusion has, therefore, been drawn from the limited data of the early effects of CD24 on radiation exposure to these lines.

It has not escaped us that a study using monoclonal antibody treatment against CD24 induced HNSCC tumors along with cisplatin treatment in athymic mice can throw light upon the additive effects of targeting CD24. Also, time staggered delivery, simultaneous delivery or a sequential application of anti-CD24 treatment and cisplatin delivery can further help understand the mechanics of CD24 expression and cisplatin resistance in HNSCC tumors in vivo. Currently, cisplatin treatment in advanced laryngeal carcinoma is directed towards eradicating the tumor bulk, but, clearly, the residual resistant population remains and causes the relapse. Our study identifies CD24+ cells as a major feature of the residual resistant population. Our study also highlights the functional relevance of CD24 positivity in sustaining a highly self-renewing, resilient population. Therefore, these results demonstrate the attractiveness of further studying and utilizing CD24 as a predictive indicator and a therapeutic target for treatment of head and neck cancers.
